# Cross-cultural translation and content validity of the Determinants of Physical Activity Questionnaire (DPAQ) in a Dutch stroke rehabilitation population and their peers without stroke

**DOI:** 10.1186/s41687-026-01058-5

**Published:** 2026-04-17

**Authors:** Desi C. M. Stokman-Meiland, Aleid de Rooij, Natalie Taylor, Sarah van Lierde, Rienk Dekker, Thea P. M. Vliet Vlieland, Monique A. M. Berger

**Affiliations:** 1 Department of Rehabilitation Medicine, University Medical Centre Groningen, University of Groningen, Groningen, The Netherlands; 2 Basalt, Vrederustlaan 180, 2543 SW, The Hague, The Netherlands; 3 Department of Orthopaedics, Rehabilitation and Physical Therapy, Leiden University Medical Centre, Leiden, The Netherlands; 4 Implementation to Impact (i2i); School of Population Health, Faculty of Medicine, UNSW Sydney, Australia; 5 Research Group Assistive Technology for Mobility & Sports, The Hague University of Applied Sciences, The Hague, The Netherlands

**Keywords:** Translation, Content validity, DPAQ, Determinants, Physical activity, Stroke

## Abstract

**Background:**

Stroke patients are less physically active than the general population. Various determinants can affect post-stroke physical activity; understanding these determinants can help tailor targeted interventions. The Determinants of Physical Activity Questionnaire (DPAQ), based on the Theoretical Domains Framework, assesses determinants of physical activity. This study aimed to translate the DPAQ into Dutch and evaluate its content validity (comprehensibility, relevance and comprehensiveness) in a stroke population and their peers without stroke.

**Methods:**

The DPAQ was translated according to international guidelines. Content validity was assessed at an outpatient neurorehabilitation clinic. Seven stroke patients completed the DPAQ using think aloud and cognitive debriefing. Audio-recorded data were transcribed, coded and analysed. The DPAQ was adapted and re-tested in seven other stroke patients and seven peers.

**Results:**

The translation and content validity test resulted in the following adaptations: the DPAQ introduction was clarified; the response option ‘Do not want to answer’ was changed to ‘Cannot answer’; and some negatively phrased items were converted to positive. Of the adapted version, most items were found comprehensible and relevant by both stroke patients and peers, except for items on coping planning (complex sentence structure and abstract item-wording) and goal conflict (irrelevant for some patients and peers). The DPAQ was found comprehensive for both patients and peers.

**Conclusions:**

A Dutch version of the DPAQ was created, which was considered comprehensible, relevant and comprehensive for both stroke patients and peers without stroke.

**Supplementary Information:**

The online version contains supplementary material available at 10.1186/s41687-026-01058-5.

## Background

Stroke patients are less physically active than the general population [1, 2]. In stroke patients, physical activity is particularly important because besides preventing major health problems [3–5], it may also improve post-stroke outcomes, including mobility, daily functioning, cognition and mental wellbeing [6–8]. Whether a stroke patient engages in physical activity may be influenced by a complex interplay of determinants (i.e. barriers and facilitators), which are likely multifactorial and vary between individuals [9]. Identifying these determinants at the individual level is important.

To identify physical activity determinants in individual stroke patients, several Patient Reported Outcome Measures (PROMs) exist [10–14]. These PROMs contain items on stroke-related (e.g. locomotor problems, fatigue, accessibility of facilities) and generic determinants (e.g. knowledge, motivation, social support) [10–14]. However, none of these PROMs is based on a theoretical model of behavioural change, and only one is informed by the ecological framework, an overarching framework that identifies the levels at which behavioural determinants operate (e.g., intrapersonal, organisational) [13]. Using a theoretical model or framework is important because it helps to identify determinants that may influence behaviour, thereby substantiating the development of targeted, potentially more effective interventions [15, 16].

The wide variety (> 80) of theoretical models of behaviour and behavioural change [17], which range in scope but often overlap, indicates that relying on only one theoretical model risks overlooking relevant determinants during intervention development [18]. The Theoretical Domains Framework (TDF) addresses this by integrating 33 theorical models into 11 domains [18] (later refined to 14 domains [19]), thereby enabling a comprehensive analysis of determinants of behaviour. The TDF has been used in questionnaire studies [20]. However, to our knowledge, the only validated TDF-based PROM on determinants of physical activity is the English-language Determinants of Physical Activity Questionnaire (DPAQ), comprising 11 domains and 34 items. It has shown promising reliability and validity in university staff and students [21], but is not available in Dutch, has not yet been studied in stroke patients and content validity has not previously been established.

Content validity (i.e. comprehensibility, relevance and comprehensiveness) is considered a crucial measurement property for PROMs [22]. In stroke patients, PROM completion may be hampered by cognitive or communicative problems [23, 24] and stroke-related determinants may differ from determinants of physical activity in the general population [9]. Consequently, the DPAQ may have to meet different requirements for stroke patients than for peers without stroke regarding comprehensibility, relevance and comprehensiveness.

Therefore, the first aim of this study was to translate the English DPAQ cross-culturally into Dutch and to make adaptations to make it suitable for use in the outpatient stroke rehabilitation population. The second aim was to evaluate the content validity of the translated and adapted DPAQ in an outpatient stroke rehabilitation population and their peers without stroke.

## Methods

### Design

This study consisted of two stages: (1) Translation of the original English DPAQ cross-culturally into Dutch and make adaptations to make it suitable for the outpatient stroke rehabilitation population; (2) Evaluation of its content validity using thinking aloud and cognitive debriefing. Content validity was determined in two rounds. The first round was conducted with stroke patients. Hereafter, the DPAQ was modified where necessary. To arrive at a final Dutch DPAQ, the modified Dutch DPAQ was retested in a second round in another sample of stroke patients and their peers without stroke.

The study was approved by the Medical Research Ethics Committee Leiden Den Haag Delft (METC LDD no. N22.083). The METC declared that the study protocol did not fall under the scope of the Medical Research Involving Human Subjects Act, that is part of the Dutch law. All study procedures were executed in accordance with the Helsinki Declaration of 1975, as revised in 2013 [25]. All participants gave their written informed consent prior to study participation.

### The DPAQ

The original English DPAQ consists of 34 items, organised into 11 domains. Items are scored using a 7-point Likert scale ranging from 1 = strongly disagree to 7 = strongly agree [21]. The DPAQ includes some negatively phrased items that should be scored inversely. The response option ‘Do not want to answer’ is scored as missing value. Mean domain scores can be computed by averaging the scores of the items belonging to the domain. A lower mean domain score indicates a higher perceived barrier.

### Stage 1: Translation

The DPAQ was translated cross-culturally into Dutch according to international guidelines by Beaton et al. [26]. Words were formulated as much as possible at level B1 according to the Common European Framework [27, 28]. First, two bilingual native Dutch speakers independently performed a forward translation from English into Dutch. Next, the two forward translations (T1 and T2) were compared; discrepancies were resolved by discussion between the translators and first author (DS), resulting in a single consensus version (T12). Subsequently, the consensus version (T12) was back translated into English by two independent bilingual native English speakers. All discrepancies between the back translations and original DPAQ were discussed by an expert committee, consisting of the first author (DS), the original developer of the DPAQ (NT), the four translators, a language professional, a methodologist (TV), a physiotherapist, movement therapist, psychologist and senior research group members (AdR, MB). Consensus was reached on final wording, grammatical issues, cultural relevance and suitability for stroke patients who attend(ed) outpatient rehabilitation, resulting in the pre-final Dutch DPAQ. During all steps, a written report of all considerations and decisions was made and thoroughly discussed in the research group (DS, AdR, MB) to draw conclusions and decide on the next steps.

### Stage 2: Evaluation of content validity

**Table 1:** Characteristics of participants

	Stroke patients first round *N* = 7)	Stroke patients second round (*N* = 7)	Peers without stroke second round (*N* = 7)
Maximum time between completion of baseline characteristics questionnaires and think-aloud interview, days, median (range)	15 (5–24)	8 (1–33)	19 (1–39)
*Sociodemographic characteristics*			
Sex, male, N	3	6	1
Age, years, median (range)	54 (42–76)	65 (29–70)	60 (33–71)
Education level*, N			
Low	4	0	1
Medium	2	4	2
High	1	3	4
Living status, alone, N	1	2	1
Employment, yes, N	4	3	5
*Stroke related characteristics*			
First stroke, N	5	4	n.a.
Type of stroke, ischemic, N	5	4	n.a.
Localization of stroke, N			
Left hemisphere	1	3	n.a.
Right hemisphere	2	2	n.a.
Other**	4	2	n.a.
Time since stroke, months, median (range)	9 (3–13)	8 (3–14)	n.a.
smRSq score, N			
0	1	0	n.a.
1	4	3	n.a.
2	1	2	n.a.
3	1	2	n.a.
4	0	0	n.a.
5	0	0	n.a.
SIS cognition domain, median (range)	82 (46–86)	82 (54–100)	n.a.
*Rehabilitation-related characteristics*			
Time between start of outpatient rehabilitation and study participation, months, median (range)	4 (0–8)	3 (2–8)	n.a.
*Health related characteristics*			
Able to walk independently, yes, N	7	7	7
Mobility aid, N	1	5	0
Communication problems			
Difficulty with expression, yes, N	1	3	0
Difficulty with understanding, yes, N	1	3	0
Expressive aphasia, yes, N	n.a.	2	n.a.
Token test score, median (range)*** *N* = 2	n.a.	29 (28–30)	n.a.
Receptive aphasia, yes, N	n.a.	0	n.a.
Speech apraxia, yes, N	n.a.	1	n.a.

n.a.: not assessed; * low: ≤ lower technical and vocational training; medium: > low and ≤ secondary technical and vocational training; high: > medium, including higher technical and vocational training and university; ** Other = cerebellar (first round N = 2), bilateral (first round N = 1), subarachnoidal (first round N = 1), brain stem (second round N = 2); *** average of 2.5 months between token test and inclusion in the study, with subjective improvement reported by speech therapist over this period for both patients

#### Setting and participants

This study was conducted at the outpatient neurorehabilitation clinics of Basalt Leiden and The Hague in the Netherlands between December 2022 and April 2023. Patients were selected by their treating physician (or a delegate) based on the following eligibility criteria: recurrent or first ever stroke, age ≥ 18 years, and able to move independently indoors (walking or in a wheelchair). Patients who were completely dependent on an electric wheelchair for locomotion indoors or who were unable to communicate in Dutch due to insufficient command of the language, cognitive impairments and/or aphasia, making an interview or PROM completion impossible, were excluded. Insufficient variability in patient characteristics in the first round, prompted selective recruitment in the second. Eligible patients were approached by the investigator by phone and received written information if interested.

To assess whether peers without stroke perceived other content validity issues than stroke patients, eligible patients from the first and second round were asked to propose a peer without stroke (i.e. partner, relative and/or friend). Proposed peers were contacted by phone, screened for eligibility and received written information if eligible and interested. Eligibility criteria for peers were: no history of stroke, age ≥ 18 years, able to move independently indoors (walking or in a wheelchair), not completely dependent on an electric wheelchair for locomotion indoors and able to communicate in Dutch.

Based on COSMIN guidelines and existing literature [22, 29], it was expected that seven patients in the first round and seven patients and seven peers without stroke in the second round would be sufficient to reach data saturation.

#### Data collection

##### Sociodemographic characteristics

For stroke patients, age (years) and sex (female/male) were extracted from the medical files. Both stroke patients and peers without stroke completed online questionnaires on basic sociodemographic characteristics (Table 1).

##### Health-related characteristics

Stroke patients and peers without stroke completed the same online questionnaires on health-related characteristics. Motor impairments were questioned and reported as ‘the ability to walk independently’ (yes/no) and ‘the usage of mobility aids’ (yes/no). Communication problems were measured using two self-developed questions: ‘Do you have difficulty expressing yourself, such as speaking and writing?’ (yes/no) and ‘Do you have difficulty understanding spoken and written language?’ (yes/no).

##### Stroke-related and rehabilitation-related characteristics

For stroke patients, the following characteristics were extracted from the medical files: type (ischemic/haemorrhagic) and localization (left hemisphere/right hemisphere/other) of stroke, first or recurrent stroke, time since stroke (months), and time between start of outpatient rehabilitation and study participation (months). For patients with mild aphasia, results on the last shortened Token Test and whether patients had expressive aphasia, receptive aphasia or speech apraxia were also collected from the medical files, and if in doubt, the attending speech therapist was contacted. All stroke patients completed an online questionnaire on stroke severity, namely the simplified modified Rankin Scale questionnaire (smRSq) resulting in an ordinal scale score ranging from 0 (no symptoms) to 5 (severe disability) [30]. Self-reported cognitive functioning was measured using the Stroke Impact Scale (SIS) 3.0 cognition domain [31] containing seven items rated in a 5-point Likert scale ranging from ‘extremely difficult’ to ‘not difficult at all’. A summative cognitive domain score was calculated, ranging from 0 to 100, with higher scores indicating better self-reported cognition.

##### DPAQ

Within three weeks of completing baseline characteristics questionnaires, participants completed the pre-final Dutch DPAQ using the think aloud method [32] at the rehabilitation centre. Participants verbalised their thoughts during completion and obvious difficulties or hesitations were registered by the researcher. Directly after completion, a cognitive debriefing interview [22, 29, 32] using a semi-structured interview guide based upon ISPOR guidelines [33] was conducted by a trained researcher (SvL) and lasted approximately one hour. The interview guide could be iteratively improved when necessary. Questions addressed the comprehensibility of the DPAQ introduction and items; and the relevance of the DPAQ items and response options. Comprehensiveness was assessed by asking participants whether they experienced barriers that were not addressed in the DPAQ and whether they were missing any questions regarding barriers to physical activity. Participants were encouraged to comment on (observed) difficulties or unclear wording.

#### Data analyses

Participant characteristics were analysed using IBM SPSS version 25 (IBM Corp: Armonk, NY, USA 2013) and described using descriptive statistics (median (range) or number).

All think aloud and cognitive debriefing interviews were audio recorded and transcribed verbatim. Analyses were performed using Atlas.ti 22. Two researchers (DS and SvL) independently coded the transcripts by labelling fragments against the three areas of content validity: comprehensibility, comprehensiveness and relevance (Table 2). Comprehensibility was divided into two subcodes: non-item-specific and item-specific. Item-specific comprehensibility was again divided into several subcodes inspired by Rausch et al. [34]: double-barrelled questions; complex sentence structure; ambiguity of items; specificity of item-wording; vocabulary; and use of jargon. The subcodes ‘example’ and ‘negative items’ were added because the DPAQ contains examples and negative items that could potentially lead to item-specific comprehensibility problems. Relevance of items and relevance of response options were separately coded. Any discrepancies in coding were discussed by the two researchers (DS and SvL) and, where necessary, presented to a third researcher (AdR) until they reached consensus. After reaching consensus, a report was generated per code with all related fragments. The research group reviewed these reports to determine necessary adaptations to the DPAQ. The adaptations were then emailed to the expert committee for feedback. Thereafter, the final Dutch DPAQ was established.

**Table 2:** Definitions of (sub)codes

(Sub)codes	Definition
*Comprehensibility*	
Non-item-specic comprehensibility	A non-item specific component of the questionnaire (e.g. introduction, layout) is not understood by the participant as intended.
Item-specific comprehensibility	An item is not understood by the participant as intended.
Double-barreled questions	One (seemingly) item contains two issues, and it’s impossible to give a single answer to cover them.
Complex sentence structure	Complex sentence that requires the participant to read the sentence several times.
Ambiguity of items	Various participants interpret some word or phrase in different ways due to ambiguity.
Specificity of item-wording	The participant has difficulty interpreting the item or gives a striking interpretation of a word because the word can be interpreted broadly.
Vocabulary	Word is not known by the participant.
Use of jargon	Specialized language used by people in the same work or profession.
Example	Given example is unclear or confusing or has an undesirable major impact on response choice.
Negative items	A negative item is worded in such a way as to require a “no” response for an affirmative answer and a “yes” response for a negative answer. The participant has difficulty answering the negative item or answers incorrectly.
*Comprehensiveness*	Is the construct (perceived barriers for increasing physical activity) completely covered by the items?
*Relevance*	
*Relevance of items*	Are the items relevant for measuring the construct (perceived barriers for increasing physical activity)?
*Relevance of response options*	Whether the response option was found relevant by the participant.

## Results

### Translation

The original English DPAQ items are shown in Table 3. The two forward translations were mostly similar, with only minor discrepancies in wording, tense or sentence structure. However, one item (Mg2) posed a cross-cultural translation problem, as there is no exact Dutch equivalent for ‘I *CANNOT be bothered* to do physical activity’. In addition, in 11 items (Kn1, En2, En4, Sk4, Sk5, Ap1, Ap2, Ap3, Ap4, Cp2, Cp6), discrepancies were caused by linguistic translation differences due to multiple possible synonyms in Dutch, for example regarding *‘to a good enough standard’* and *‘work around obstacles’*. Discrepancies were discussed, resulting in a consensus version (T12) after forward translations.

**Table 3:** Original English version and final Dutch version of the Determinants of Physical Activity Questionnaire (DPAQ)

DPAQ domain	DPAQ item	Original English version	Final Dutch version
Knowledge	Kn1	I know what the recommended levels of physical activity are	Ik weet welke adviezen er in de Nederlandse Beweegrichtlijnen staan
	Kn2	I DO NOT know the reasons why I should be meeting the nationally recommended physical activity guidelines	Ik weet waarom ik aan de Nederlandse Beweegrichtlijnen zou moeten voldoen
	Kn3	I have NOT previously read information about the current nationally recommended physical activity guidelines	Ik heb nog NOOIT van de Nederlandse Beweegrichtlijnen gehoord
Environmental resources	En1	Facilities are available to help me to do physical activity	Er zijn voorzieningen in mijn buurt beschikbaar die mij helpen om aan lichaamsbeweging te doen
	En2	There is NO WHERE to do physical activity near me	Er is bij mij in de buurt GEEN plek waar ik aan lichaamsbeweging kan doen
	En4	My local area is NOT very attractive and this puts me off doing physical activity	Mijn buurt is NIET erg aantrekkelijk en dit houdt mij tegen om aan lichaamsbeweging te doen
Motivation and goals	Mg1	I want to do physical activity	Ik wil aan lichaamsbeweging doen
	Mg2	I CANNOT be bothered to do physical activity	Ik heb GEEN interesse om aan lichaamsbeweging te doen
	Mg3	I feel motivated to do physical activity	Ik ben gemotiveerd om aan lichaamsbeweging te doen
Beliefs about capabilities	Bcap1	I DO NOT feel confident when doing physical activity	Ik voel mij ONZEKER als ik aan lichaamsbeweging doe
	Bcap2	Doing physical activity makes me feel embarrassed	Ik schaam mij wanneer ik aan lichaamsbeweging doe
	Bcap3	I FIND IT HARD to do physical activity when I see others doing well at physical activity (e.g. watching others run for a long time on the treadmill)	Ik vind het MOEILIJK om aan lichaamsbeweging te doen als ik zie hoe goed anderen dat doen
Emotion	Em4	Daily life is too stressful for physical activity	Het dagelijks leven is te stressvol om aan lichaamsbeweging te doen
	Em5	I have too many negative emotions which prevent me from doing physical activity	Te veel negatieve emoties houden mij tegen om aan lichaamsbeweging te doen
	Em6	When I think about doing physical activity, I start to worry	Als ik denk aan het doen van lichaamsbeweging, begin ik mij zorgen te maken
Skills	Sk4	I can do physical activity to a good enough standard	Ik heb genoeg lichamelijke vaardigheden om aan lichaamsbeweging te doen
	Sk5	I’ve NEVER really had sports skills so I DON’T do physical activity	Ik ben nooit goed in sport geweest, dus ik doe NIET aan lichaamsbeweging
	Sk6	I don’t seem to have the skills to keep going in physical activity sessions	Als ik aan lichaamsbeweging doe kan mijn lichaam dat NIET lang volhouden
Social influences	Si3	My friends DON’T support or encourage my physical activity	Mijn vrienden steunen mij NIET of moedigen mij NIET aan om aan lichaamsbeweging te doen
	Si6	The people I spend my free time with don’t do physical activity	De mensen met wie ik mijn vrije tijd doorbreng doen NIET aan lichaamsbeweging
	Si7	I DON’T have anyone to do physical activity with	Ik heb NIEMAND met wie ik aan lichaamsbeweging kan doen
Beliefs about consequences	Bcon1	If I do PA, it will benefit me in the short term (e.g. burn calories, sleep better etc.)	Als ik aan lichaamsbeweging doe, heeft dit op de korte termijn voordelen voor mij (bijvoorbeeld calorieën verbranden, beter slapen, enzovoort)
	Bcon2	If I do PA it will benefit me in the long term (e.g. live longer, lose weight etc.)	Als ik aan lichaamsbeweging doe, heeft dit op de lange termijn voordelen voor mij (bijvoorbeeld langer leven, afvallen, enzovoort)
	Bcon4	I think physical activity will change my life for the better	Ik denk dat lichaamsbeweging mijn leven positief verandert
Action planning	Ap1	I tend to plan where my PA will happen (e.g. at the park, leisure centre etc.)	Ik bedenk meestal van tevoren waar ik aan lichaamsbeweging ga doen (bijvoorbeeld in het park, in de sportschool, enzovoort)
	Ap2	I do not tend to plan when my PA will happen (e.g. Monday at 6pm etc.)	Ik plan meestal wanneer ik aan lichaamsbeweging ga doen (bijvoorbeeld maandag om 18:00 uur, enzovoort)
	Ap3	I tend to plan how my PA will happen (e.g. how to get there, kit needed etc.)	Ik bedenk meestal van tevoren wat ik nodig heb om aan lichaamsbeweging te doen (bijvoorbeeld hoe ik ernaartoe zal gaan, welke spullen ik nodig heb, enzovoort)
	Ap4	I do not tend to plan what type of PA I will do (e.g. aerobics class, walking to work, session at the gym etc.)	Ik plan meestal welk soort lichaamsbeweging ik zal gaan doen (bijvoorbeeld wandelen, fietsen, sportschool, enzovoort)
Coping planning	Cp2	I know what to do in difficult situations in order to make sure I do the physical activity I have planned	Ik weet wat ik op moeilijke momenten kan doen om de door mij geplande lichaamsbeweging toch uit te voeren
	Cp5	I get easily distracted from the physical activity I have planned	Wanneer ik van plan ben om aan lichaamsbeweging te gaan doen laat ik daar makkelijk dingen tussenkomen
	Cp6	I always work around obstacles to physical activity; nothing really stops me	Ik vind altijd een oplossing voor dingen die mij tegenhouden om aan lichaamsbeweging te doen: niets houdt mij tegen
Goal conflict	Gc1	I WOULD NOT be prepared to give up work ambitions to do physical activity	Ik zou mijn werkambities op willen geven om aan lichaamsbeweging te doen
	Gc2	I would be prepared to give up things I usually do in my leisure time for physical activity	Ik zou dingen die ik in mijn vrije tijd doe op willen geven om aan lichaamsbeweging te doen
	Gc3	I WOULD NOT be prepared to give up spending time with my friends for physical activity	Ik zou tijd die ik doorbreng met mijn vrienden op willen geven om aan lichaamsbeweging te doen

After backward translation, the expert committee discussed the original DPAQ and translations. Regarding the DPAQ introduction, they felt that, for stroke patients attending outpatient rehabilitation, the context of use and recall period were unclear. Therefore, the recall period (at this moment rather than pre-stroke) and context of use (home environment rather than rehabilitation setting) were added. Additionally, the DPAQ topic (determinants of physical activity) was not mentioned in the introduction and the target behaviour (physical activity) was insufficiently specified. Therefore, determinants of physical activity (‘things that help or hinder you’); a specified target behaviour (‘physical activity of all intensities’); and examples of this target behaviour (e.g. cycling, dressing) were added.

The expert committee made noticeable adjustments to the consensus version (T12) in eight items (Kn1, En1, Sk4, Sk6, Ap1, Ap3, Ap4, Cp5) (Table 4), in close consultation with the original developer. For example, ‘skills’ were clarified to ‘physical skills’ (Sk4, Sk6) and ‘I get easily distracted from’ (Cp5) was changed to ‘I easily let something get in between’ because this better approximated the developer’s intent. Minor adjustments were made, for example, to improve the natural readability of items in Dutch.

**Table 4:** Noticeable adjustments made by the expert committee to items of the consensus version after forward translations of the Dutch DPAQ

DPAQ item*	Problem description	Adjustment made
Kn1	Unlike the other two items from the knowledge domain, this item does not refer to the Dutch physical activity guidelines	Clarified by referring to the Dutch physical activity guidelines in line with the other items from the knowledge domain
En1	Unlike the other two items from the environmental resources domain, this item does not refer to ‘in my neighbourhood’	Clarified by adding ‘in my neighbourhood,’ in line with the other items from the environmental resources domain
Sk4	Due to multiple possible synonyms in Dutch, choosing the translation that best approximated the original developer’s intent was challenging	In close consultation with the original developer, the Dutch translation of ‘I have enough physical skills to engage in physical activity’ was chosen because this best approximated the developer’s intent
Sk6	‘The skills to keep going in physical activity sessions’ can imply both physical and mental skills	In close consultation with the original developer, ‘skills’ were clarified to ‘physical skills,’ including physical fitness
Ap1	‘To plan’ in Dutch does not apply to ‘where’	‘To plan where’ was changed to ‘to consider in advance where’
Ap3	‘To plan’ in Dutch does not apply to ‘how’	‘To plan how’ was changed to ‘to consider in advance how’ and ‘How physical activity will happen’ was adjusted to ‘what is needed to do physical activity’ to create a proper Dutch sentence that best approximates the original developers’ intent
Ap4	The item contained examples of physical activity that are not commonly known in the Netherlands (e.g. aerobics class)	Examples were modified to suit the Dutch context
Cp5	The Dutch translation of ‘I get easily distracted from’ did not match the original translator’s intent	In close consultation with the original developer the Dutch translation of ‘I easily let something get in between’ was chosen because this better approximated the developer’s intent

DPAQ = Determinants of Physical Activity Questionnaire * Original English version and final Dutch version of the DPAQ items are presented in Table 3

The pre-final Dutch DPAQ was established hereafter. A written report was made of all considerations and decisions.

### Evaluation of content validity

#### First round

Table 1 presents participant characteristics. All seven patients (median age 54; almost half male) were able to walk independently, one patient used an assistive walking device. One patient reported communication problems. The median SIS cognition domain score was 82 (range 46–86).

Several patients summarized the introduction to the DPAQ differently than intended and instructions on recall period and context of use were not consistently applied. Therefore the research group concluded that the introduction was not comprehended as intended and hypothesized that length and complex sentences may have contributed to this. In addition, the research group found that the target behaviour (physical activity of all intensities) was improperly chosen because patients indicated they mainly thought about moderate-intensity physical activities while completing the DPAQ. Adjustments to the introduction were made to address these issues.

Most of the items were well comprehended, except for five negatively phrased items, all coping planning items (due to a complex sentence structure, a double barrelled question and abstract item-wording) and the items Bcap3 (example not recognized by some patients) and Gc2 (ambiguity of item-wording sometimes led to confusion about the recall period) (Table 5). Those items were adapted to resolve the problems mentioned, although the research group suspected that the abstract wording of the coping planning items could not be fully resolved.

**Table 5:** Item-specific comprehensibility problems of the pre-final Dutch DPAQ and adaptations made

DPAQ item*	Item-specific comprehensibility problem	Adaptation made
*Evaluation of content validity – first round (stroke patients)*		
Kn2	Negative item – led to comprehensibility problems in some stroke patients	Negative item was converted to positive item
BCap3	Example - some patients did not recognize themselves in the example used	the example “ watching others run for a long time on the treadmill” was omitted
Ap2	Negative item – led to comprehensibility problems in some patients	Negative item was converted to positive item
Ap4	Negative item – led to comprehensibility problems in some patients	Negative item was converted to positive item
Cp2	Complex sentence structure	Sentence structure was simplified
Cp5	Specificity of item wording - responses tended to reflect a specific situation the patient had in mind	Singular words were modified to plural to prompt consideration of multiple obstacles and how to cope with them. The abstract wording could not be fully resolved.
Cp6	Double-barrelled question	Sentence structure was simplified by removing ‘nothing really stops me’.
	Specificity of item wording - responses tended to reflect a specific situation the patient had in mind	Singular words were modified to plural to prompt consideration of multiple obstacles and how to cope with them. The abstract wording could not be fully resolved.
Gc1	Negative item – led to comprehensibility problems in some patients	Negative item was converted to positive item
Gc2	Ambiguity of item – led to confusion about the recall period in some stroke patients	The word ‘usually’ was omitted since that word could not be unambiguously translated to Dutch and therefore led to confusion about the recall period in some stroke patients (at this moment or before stroke)
Gc3	Negative item – led to comprehensibility problems in some patients	Negative item was converted to positive item
*Evaluation of content validity – second round (stroke patients and peers without stroke)*		
BCap1	Negative item – led to comprehensibility problems in some stroke patients and peers without stroke	‘I do not feel confident’ was changed to ‘I feel insecure’ to eliminate the negative phrasing (‘not’)
Cp2	Complex sentence structure	Other words were chosen to avoid repetition of words
Cp6	Complex sentence structure	The item was restored to its double-barrelled form to remain more consistent with the original item

DPAQ = Determinants of Physical Activity Questionnaire * Original English version and final Dutch version of the DPAQ items are presented in Table 3

Most patients reported that the DPAQ was comprehensive, although some doubted whether fatigue and lack of energy were adequately reflected. Since the DPAQ does not explicitly question stroke-specific determinants, the research group found it remarkable that most stroke patients considered the DPAQ comprehensive. Therefore, for the second round, the interview guide was adapted and participants were asked to list their top three barriers to physical activity. Based on the barriers listed, the research group assessed the comprehensiveness of the DPAQ.

No relevance issues were reported by patients regarding most DPAQ items; only the relevance of goal conflict items (Gc1, Gc2) was discussed in the research group. Responses on these items did not always adequately reflect whether the ‘goal conflict’ was a barrier to physical activity, for example among the following patients: patients who did not currently work; patients who had sufficient time to engage in both physical activity and work/leisure time; and patients who engaged in physical activity during work/leisure time. To solve this problem, the items would have to be completely rephrased; this was however not done as such modifications would result in too drastic deviations from the original validated version.

Finally, two patients stated that they would never choose the response option ‘Do not want to answer’. One patient used ‘Do not want to answer’ to a question about the Dutch physical activity guidelines (Kn1) because he was unfamiliar with these guidelines and to a question about coping planning (Cp2) because he found it too difficultly phrased. The research group concluded that using ‘Do not want to answer’ was not related to not wanting to answer, but to not being able to answer. Therefore, ‘Do not want to answer’ was changed to ‘Cannot answer’.

#### Second round - stroke patients

Because patients who had mild aphasia or were mobility aid-dependent were underrepresented in the first round, they were selectively selected for the second round. Table 1 displays participant characteristics. Among the seven patients (median age 65; predominantly male), mild aphasia (a score of ≤ 30 on the last shortened Token Test [35, 36]) and usage of mobility aids were frequently reported (*n* = 3 and *n* = 5, respectively).

No significant comprehensibility issues were found regarding the DPAQ introduction. However, the recall period varied among patients and among items; the instruction ‘at this moment’ was not always met. In addition, some patients associated certain items with the rehabilitation setting rather than the home environment. Finally, patients mostly used moderately intensive physical activities as a reference frame for the target behaviour. No adjustments were made for any of these issues.

Most items were considered comprehensible, except for one negative item (Bcap1), and two coping planning items (complex sentence structure) which had already been modified in the first round (Cp2, Cp6). These three items were therefore again slightly modified (Table 5). However, the research group believed that a complex sentence structure and abstract item-wording still remained in both coping planning items.

The DPAQ as a whole was considered comprehensive by the research group; all barriers mentioned by patients were considered to be captured in the DPAQ. The relevance issues regarding goal conflict items reported in the first round were also found in the second round. Again, no adaptations were made.

The response option ‘Cannot answer’ was chosen once or more by three patients, mainly regarding items of the domains knowledge and coping planning. To ensure that respondents who had never heard of the Dutch physical activity guidelines will answer appropriately, the following instruction was added: ‘if you cannot answer the question because you have never heard of the Dutch physical activity guidelines, please fill in ‘completely disagree’’.

#### Second round – peers without stroke

Table 1 displays participant characteristics (median age 60, predominantly female). The DPAQ introduction was well comprehended by peers without stroke. However, as in stroke patients, the recall instruction was not always met, for which no adjustments were made.

Most items were comprehensible to peers without stroke; only the same items as in stroke patients caused comprehensibility problems (i.e. one negative item (Bcap1) and all coping planning items). The research group considered the DPAQ comprehensive regarding determinants of physical activity, as it appeared to capture all barriers mentioned by peers without stroke.

Similar to stroke patients, some peers seemed to encounter relevance issues regarding the goal conflict item about work (Gc1). No relevance issues were reported regarding other DPAQ items. The response option ‘Cannot answer’ was chosen once or more by two peers, including: once regarding a knowledge item, once regarding a coping planning item and twice regarding goal conflict items.

The translation, adaptation and content validation process finally resulted in a final Dutch DPAQ, which is available in Table 3 and Appendix 1.

## Discussion

In this study, the original English DPAQ, a 34-item 11-domain PROM measuring determinants of physical activity, was translated into Dutch according to international standards. The Dutch DPAQ shows promising content validity (i.e. comprehensibility, relevance and comprehensiveness) in both stroke patients and peers without stroke. The Dutch DPAQ allows a comprehensive analysis of determinants of physical activity based on the TDF and thereby can provide valuable information for targeting interventions.

To our knowledge, the DPAQ is the only available PROM on determinants of physical activity based on the TDF. This study is the first to cross-culturally translate the DPAQ and evaluate its content validity, one of the most important measurement properties of a PROM [22], and the first to examine this PROM in stroke patients.

During the translation and content validity process, some adjustments were made to the DPAQ. The introduction was slightly adapted to specify the target behaviour, context of use and recall period. The adaptations regarding the target behaviour align with the Behaviour Change Wheel (BCW) [37, 38], which emphasises the importance of specifying the target behaviour, among other things to facilitate interpretation of the determinants of the target behaviour [20]. The context of use and recall period were added as a clear context of use and recall period are important for content validity [22]. The adjustments mentioned above should improve the (content) validity and reliability of the DPAQ; cross-cultural validity [39] may be negatively affected.

Our finding that instructions on context of use and recall period were not consistently applied aligns with previous research showing that instruction on recall period may not be enough; the wording and tense of items may need adjustment to obtain a specific recall period [40]. To avoid the complex process of adapting and retesting items, the context of use and recall period might be repeated above each item (or page), helping both stroke patients, especially those with cognitive problems, and peers without stroke to adhere to those instructions. For stroke patients, adding ‘post-stroke’ to the recall period (i.e. at this moment post-stroke) may be considered.

Another adaptation done to the DPAQ was that negatively phrased items causing comprehensibility problems were reworded positively. Previous research already showed that negative sentences require a longer reaction time [41], which may be exacerbated in stroke patients with cognitive problems, and thereby may increase the burden of PROM completion. Although the use of both negatively and positively phrased items in a PROM is advised by some authors to minimize acquiescent and extreme response bias [42, 43], there is insufficient evidence that the advantages outweigh the disadvantages [44–46], justifying our decision to reword some negatively phrased items positively.

Finally, the response option ‘Do not want to answer’ was changed to ‘Cannot answer’ as ‘Do not want to answer’ was considered irrelevant by some patients, probably because physical activity is not a sensitive topic. ‘Cannot answer’ seems to fulfil a need, as it was chosen regularly. However, since this response option must be scored as missing, it may cause (perhaps unnecessarily) missing data. The advantages and disadvantages of the ‘Cannot answer’ response option should be carefully considered in the further development of the DPAQ.

Strengths of this study were that international guidelines were followed during the translation process [26]; the original developer of the DPAQ (NT) was closely engaged; an expert committee with a wide variety of expertise was involved; a written report was made of all findings and considerations; and all those findings and considerations were discussed extensively by the research group before being included in this article.

This study also had some limitations. First, sample size was based on COSMIN recommendations rather than formal assessment of data saturation. However, according to COSMIN guidelines, a sample of ≥ 7 participants is considered ‘very good’ [22], and the gradual decline in the number of newly identified content validity issues suggests that most relevant issues were captured. Second, despite the inclusion of patients with mild aphasia, the DPAQ may not be suitable for patients with moderate/severe aphasia or moderate/severe cognitive problems. Further research and development, such as the work by Jensen et al. [47], is needed to adapt the PROM for use in these patients. Third, all participants were able to walk independently. Patients unable to walk independently, may have other requirements regarding relevance and comprehensiveness of the DPAQ. However, based on this study, we cannot comment on this. Fourth, although an appropriate method to determine comprehensiveness was used in the first round, the research group doubted whether the abstract reasoning required to judge comprehensiveness was feasible for patients [47]. Therefore, in the second round, the research group assessed comprehensiveness based on patient-reported barriers to physical activity, a method that has its own disadvantages, such as potential bias due to researcher interpretation. In addition, the focus on barriers may be questionable, since facilitators are also important determinants of behavioural change [20, 48]. Further research on comprehensiveness is recommended, including reconsidering the methodology for determining comprehensiveness. Finally, although the replacement of ‘Do not want to answer’ with ‘Cannot answer’ has been carefully considered, ‘Cannot answer’ is significantly different from ‘Do not want to answer’. Further exploration is needed to determine whether this modification is desired.

The results of this study are promising regarding the use of the Dutch DPAQ in clinical practice. The DPAQ may provide a solid foundation for initiating a conversation on determinants of physical activity with a healthcare professional [49, 50]. Given the diversity and potential interrelatedness of determinants of physical activity [9, 19] and the proven added value of discussing PROM results with a healthcare professional in order to identify patients in need of support [51], it is expected that an additional in-depth conversation on determinants of physical activity is still needed after DPAQ completion. It is suggested that such a conversation should focus primarily on perceived barriers and facilitators (i.e. lowest and highest mean DPAQ domain scores) and to explore whether these determinants are a possible target for intervention. Because some stroke patients and peers without stroke had problems with the comprehensibility of coping planning items and relevance of goal conflict items, extra attention during the conversation to these items might be advised.

For research purposes, the DPAQ may be a valuable addition to existing PROMs. This study showed promising content validity in both stroke patients and peers without stroke. The plausible broad applicability of the Dutch DPAQ may help compare determinants of physical activity across populations. Further research on comprehensiveness, construct validity and reliability can contribute to further optimisation of the Dutch DPAQ. In addition, studies exploring its usability, feasibility and applicability in clinical practice to support the conversation on determinants of physical activity between patient and healthcare professional are desirable.

## Conclusions

This study demonstrates that the Dutch DPAQ appears to be comprehensible, relevant and comprehensive for both stroke patients and peers without stroke. The Dutch DPAQ seems promising to provide insight into determinants of physical activity in stroke patients and peers without stroke. The Dutch DPAQ may provide a solid foundation for initiating a conversation on determinants of physical activity between a patient and healthcare professional.

## Supplementary Information

Below is the link to the electronic supplementary material.

Supplementary Material 1

## Data Availability

The datasets used and/or analysed during the current study are available from the corresponding author on reasonable request.

## Abbreviations

BCW: Behaviour Change Wheel
DPAQ: Determinants of Physical Activity Questionnaire
METC LDD: Medical Research Ethics Committee Leiden Den Haag Delft
PROM: Patient Reported Outcome Measure
SIS: Stroke Impact Scale
smRSq: simplified modified Rankin Scale questionnaire
TDF: Theoretical Domains Framework
