# Supplementary material for: Cross-cultural translation and content validity of the Determinants of Physical Activity Questionnaire (DPAQ) in a Dutch stroke rehabilitation population and their peers without stroke
Source: J Patient Rep Outcomes. 2026 Apr 17;10:91. doi: 10.1186/s41687-026-01058-5 (PMC13222906; doi:10.1186/s41687-026-01058-5)
Supplement: Supplementary file 1 — Supplementary Material 1 [file 41687_2026_1058_MOESM1_ESM.docx]

**Factoren van lichaamsbeweging**

Deze vragenlijst gaat over zaken die u helpen of in de weg staan om aan **matig intensieve lichaamsbeweging** te doen.

Matig intensief betekent dat de hartslag omhooggaat en u sneller gaat ademen. Praten is nog mogelijk, maar wordt wel iets moeilijker.

Hierbij kunt u denken aan huishoudelijke activiteiten, beweegactiviteiten (zoals oefeningen doen, lopen, rolstoelrijden) of sportactiviteiten **in uw eigen omgeving** (thuis of bij u in de buurt).

Klik voor elke vraag op het antwoord dat **op dit moment** het beste **bij u** past.

Vragen zijn soms positief en soms negatief gesteld, daarom is het belangrijk dat u de vragen goed leest.

| 1. Ik wil aan lichaamsbeweging doen | Helemaal oneens | Oneens | Beetje oneens | Neutraal | Beetje eens | Eens | Helemaal eens | Kan ik niet beantwoorden |
| --- | --- | --- | --- | --- | --- | --- | --- | --- |
| 2. Ik bedenk meestal van tevoren wat ik nodig heb om aan lichaamsbeweging te doen (bijvoorbeeld hoe ik er naar toe zal gaan, welke spullen ik nodig heb, enzovoort) | Helemaal oneens | Oneens | Beetje oneens | Neutraal | Beetje eens | Eens | Helemaal eens | Kan ik niet beantwoorden |
| 3. Als ik aan lichaamsbeweging doe, heeft dit op de korte termijn voordelen voor mij (bijvoorbeeld calorieën verbranden, beter slapen, enzovoort) | Helemaal oneens | Oneens | Beetje oneens | Neutraal | Beetje eens | Eens | Helemaal eens | Kan ik niet beantwoorden |
| 4. Als ik aan lichaamsbeweging doe kan mijn lichaam dat NIET lang volhouden | Helemaal oneens | Oneens | Beetje oneens | Neutraal | Beetje eens | Eens | Helemaal eens | Kan ik niet beantwoorden |
| 5. Ik heb nog NOOIT van de Nederlandse Beweegrichtlijnen gehoord | Helemaal oneens | Oneens | Beetje oneens | Neutraal | Beetje eens | Eens | Helemaal eens | Kan ik niet beantwoorden |
| 6. Ik voel mij ONZEKER als ik aan lichaamsbeweging doe | Helemaal oneens | Oneens | Beetje oneens | Neutraal | Beetje eens | Eens | Helemaal eens | Kan ik niet beantwoorden |
| 7. Ik bedenk meestal van tevoren waar ik aan lichaamsbeweging ga doen (bijvoorbeeld in het park, in de sportschool, enzovoort) | Helemaal oneens | Oneens | Beetje oneens | Neutraal | Beetje eens | Eens | Helemaal eens | Kan ik niet beantwoorden |
| 8. Ik heb GEEN interesse om aan lichaamsbeweging te doen | Helemaal oneens | Oneens | Beetje oneens | Neutraal | Beetje eens | Eens | Helemaal eens | Kan ik niet beantwoorden |
| 9. Mijn buurt is NIET erg aantrekkelijk en dit houdt mij tegen om aan lichaamsbeweging te doen | Helemaal oneens | Oneens | Beetje oneens | Neutraal | Beetje eens | Eens | Helemaal eens | Kan ik niet beantwoorden |
| 10. Te veel negatieve emoties houden mij tegen om aan lichaamsbeweging te doen | Helemaal oneens | Oneens | Beetje oneens | Neutraal | Beetje eens | Eens | Helemaal eens | Kan ik niet beantwoorden |
| 11. De mensen met wie ik mijn vrije tijd doorbreng doen NIET aan lichaamsbeweging | Helemaal oneens | Oneens | Beetje oneens | Neutraal | Beetje eens | Eens | Helemaal eens | Kan ik niet beantwoorden |
| 12. Er is bij mij in de buurt GEEN plek waar ik aan lichaamsbeweging kan doen | Helemaal oneens | Oneens | Beetje oneens | Neutraal | Beetje eens | Eens | Helemaal eens | Kan ik niet beantwoorden |
| 13. Ik schaam mij wanneer ik aan lichaamsbeweging doe | Helemaal oneens | Oneens | Beetje oneens | Neutraal | Beetje eens | Eens | Helemaal eens | Kan ik niet beantwoorden |
| 14. Ik vind altijd een oplossing voor dingen die mij tegenhouden om aan lichaamsbeweging te doen: niets houdt mij tegen | Helemaal oneens | Oneens | Beetje oneens | Neutraal | Beetje eens | Eens | Helemaal eens | Kan ik niet beantwoorden |
| 15. Mijn vrienden steunen mij NIET of moedigen mij NIET aan om aan lichaamsbeweging te doen | Helemaal oneens | Oneens | Beetje oneens | Neutraal | Beetje eens | Eens | Helemaal eens | Kan ik niet beantwoorden |
| 16. Ik weet waarom ik aan de Nederlandse Beweegrichtlijnen zou moeten voldoen | Helemaal oneens | Oneens | Beetje oneens | Neutraal | Beetje eens | Eens | Helemaal eens | Kan ik niet beantwoorden* |
| 17. Ik heb genoeg lichamelijke vaardigheden om aan lichaamsbeweging te doen | Helemaal oneens | Oneens | Beetje oneens | Neutraal | Beetje eens | Eens | Helemaal eens | Kan ik niet beantwoorden |
| 18. Ik heb NIEMAND met wie ik aan lichaamsbeweging kan doen | Helemaal oneens | Oneens | Beetje oneens | Neutraal | Beetje eens | Eens | Helemaal eens | Kan ik niet beantwoorden |
| 19. Ik ben nooit goed in sport geweest, dus ik doe NIET aan lichaamsbeweging | Helemaal oneens | Oneens | Beetje oneens | Neutraal | Beetje eens | Eens | Helemaal eens | Kan ik niet beantwoorden |
| 20. Ik ben gemotiveerd om aan lichaamsbeweging te doen | Helemaal oneens | Oneens | Beetje oneens | Neutraal | Beetje eens | Eens | Helemaal eens | Kan ik niet beantwoorden |
| 21. Er zijn voorzieningen in mijn buurt beschikbaar die mij helpen om aan lichaamsbeweging te doen | Helemaal oneens | Oneens | Beetje oneens | Neutraal | Beetje eens | Eens | Helemaal eens | Kan ik niet beantwoorden |
| 22. Ik zou mijn werkambities op willen geven om aan lichaamsbeweging te doen | Helemaal oneens | Oneens | Beetje oneens | Neutraal | Beetje eens | Eens | Helemaal eens | Kan ik niet beantwoorden |
| 23. Wanneer ik van plan ben om aan lichaamsbeweging te gaan doen laat ik daar makkelijk dingen tussenkomen | Helemaal oneens | Oneens | Beetje oneens | Neutraal | Beetje eens | Eens | Helemaal eens | Kan ik niet beantwoorden |
| 24. Als ik denk aan het doen van lichaamsbeweging, begin ik mij zorgen te maken | Helemaal oneens | Oneens | Beetje oneens | Neutraal | Beetje eens | Eens | Helemaal eens | Kan ik niet beantwoorden |
| 25. Ik weet welke adviezen er in de Nederlandse Beweegrichtlijnen staan | Helemaal oneens | Oneens | Beetje oneens | Neutraal | Beetje eens | Eens | Helemaal eens | Kan ik niet beantwoorden* |
| 26. Ik zou dingen die ik in mijn vrije tijd doe op willen geven om aan lichaamsbeweging te doen | Helemaal oneens | Oneens | Beetje oneens | Neutraal | Beetje eens | Eens | Helemaal eens | Kan ik niet beantwoorden |
| 27. Ik plan meestal wanneer ik aan lichaamsbeweging ga doen (bijvoorbeeld maandag om 18:00 uur, enzovoort) | Helemaal oneens | Oneens | Beetje oneens | Neutraal | Beetje eens | Eens | Helemaal eens | Kan ik niet beantwoorden |
| 28. Het dagelijks leven is te stressvol om aan lichaamsbeweging te doen | Helemaal oneens | Oneens | Beetje oneens | Neutraal | Beetje eens | Eens | Helemaal eens | Kan ik niet beantwoorden |
| 29. Ik plan meestal welk soort lichaamsbeweging ik zal gaan doen (bijvoorbeeld wandelen, fietsen, sportschool, enzovoort) | Helemaal oneens | Oneens | Beetje oneens | Neutraal | Beetje eens | Eens | Helemaal eens | Kan ik niet beantwoorden |
| 30. Ik weet wat ik op moeilijke momenten kan doen om de door mij geplande lichaamsbeweging toch uit te voeren | Helemaal oneens | Oneens | Beetje oneens | Neutraal | Beetje eens | Eens | Helemaal eens | Kan ik niet beantwoorden |
| 31. Ik vind het MOEILIJK om aan lichaamsbeweging te doen als ik zie hoe goed anderen dat doen | Helemaal oneens | Oneens | Beetje oneens | Neutraal | Beetje eens | Eens | Helemaal eens | Kan ik niet beantwoorden |
| 32. Als ik aan lichaamsbeweging doe, heeft dit op de lange termijn voordelen voor mij (bijvoorbeeld langer leven, afvallen, enzovoort) | Helemaal oneens | Oneens | Beetje oneens | Neutraal | Beetje eens | Eens | Helemaal eens | Kan ik niet beantwoorden |
| 33. Ik zou tijd die ik doorbreng met mijn vrienden op willen geven om aan lichaamsbeweging te doen | Helemaal oneens | Oneens | Beetje oneens | Neutraal | Beetje eens | Eens | Helemaal eens | Kan ik niet beantwoorden |
| 34. Ik denk dat lichaamsbeweging mijn leven positief verandert | Helemaal oneens | Oneens | Beetje oneens | Neutraal | Beetje eens | Eens | Helemaal eens | Kan ik niet beantwoorden |

* Indien u de vraag niet kunt beantwoorden omdat u nog nooit van de Nederlandse Beweegrichtlijnen heeft gehoord, vul dan helemaal oneens in

**Einde vragenlijst**
